# Novel cycloartane triterpenoid from *Cimicifuga foetida* (*Sheng ma*) induces mitochondrial apoptosis via inhibiting Raf/MEK/ERK pathway and Akt phosphorylation in human breast carcinoma MCF-7 cells

**DOI:** 10.1186/s13020-015-0073-6

**Published:** 2016-01-11

**Authors:** Hai-yan Sun, Bei-bei Liu, Jian-yang Hu, Li-jia Xu, Shun-wan Chan, Chi-on Chan, Daniel K. W. Mok, Dong-mei Zhang, Wen-cai Ye, Si-bao Chen

**Affiliations:** Institute of Medicinal Plant Development, Chinese Academy of Medical Sciences and Peking Union Medical College, Beijing, China; State Key Laboratory of Chinese Medicine and Molecular Pharmacology, Hong Kong Polytechnic University, Hong Kong, China; College of Pharmacy, Jinan University, Guangzhou, China

## Abstract

**Background:**

Cycloartane triterpenoids exhibited anticancer effects. This study aims to identify any potential novel anticancer cycloartane triterpenoids from *Cimicifuga foetida* L. rhizome (*Sheng ma*) and the mode of actions.

**Methods:**

Cycloartane triterpenoids were isolated from the *C. foetida* rhizome by a series of column chromatography and identified by IR, MS and NMR. Their anticancer effects on several human cancer cell lines, MCF-7, HepG2, HepG2/ADM, HeLa, and PC3, and normal human mammary epithelial cells MCF10A were investigated by colony formation and MTT assays. Morphological analysis of apoptosis induction was performed by acridine orange/ethidium bromide dual-staining and Hoechst 33258 nuclear staining. The cell-cycle profile and annexin V staining were evaluated by flow cytometry. Apoptosis were investigated by measuring changes in mitochondrial membrane potential and analyzing expression of cell cycle- and apoptosis-related proteins in MCF-7 cells by Western blotting.

**Results:**

A novel cycloartane triterpenoid, 25-*O*-acetyl-7,8-didehydrocimigenol-3-*O*-*β*-d-(2-acetyl)xylopyranoside (ADHC-AXpn), together with the known 7,8-didehydrocimigenol-3-*O*-*β*-d-xylopyranoside (DHC-Xpn) were isolated. MCF-7 growth was significantly inhibited by ADHC-AXpn in a dose- and time-dependent manner (IC_50_: 27.81 µM at 48 h; *P* = 0.004 vs. control at 25 μM for 48 h treatment), and ADHC-AXpn was selectively cytotoxic for cancerous cells (MCF-7, HepG2/ADM, HepG2 and HELA cells) based on its higher IC_50_ values for normal cells MCF10A (IC_50_: 78.63 µM at 48 h) than for tumor cells. In MCF-7 cells, ADHC-AXpn induced G_2_/M cell cycle arrest by mediating cyclin-B1, and CDK1 and its phosphorylation; and induced apoptosis through the mitochondrial-mediated apoptotic pathway, with inhibition of Akt activation. As ADHC-AXpn suppressed phosphorylation of ERK1/2, Raf and Akt proteins in MCF-7 cells, its apoptotic effect might be associated with Raf/MEK/ERK signaling and Akt activation.

**Conclusions:**

ADHC-AXpn significantly suppressed the growth of MCF-7 cells, induced mitochondrial apoptosis and cell-cycle arrest, and inhibited Raf/MEK/ERK signaling pathway and Akt phosphorylation.

## Background

**Fig. 1:**
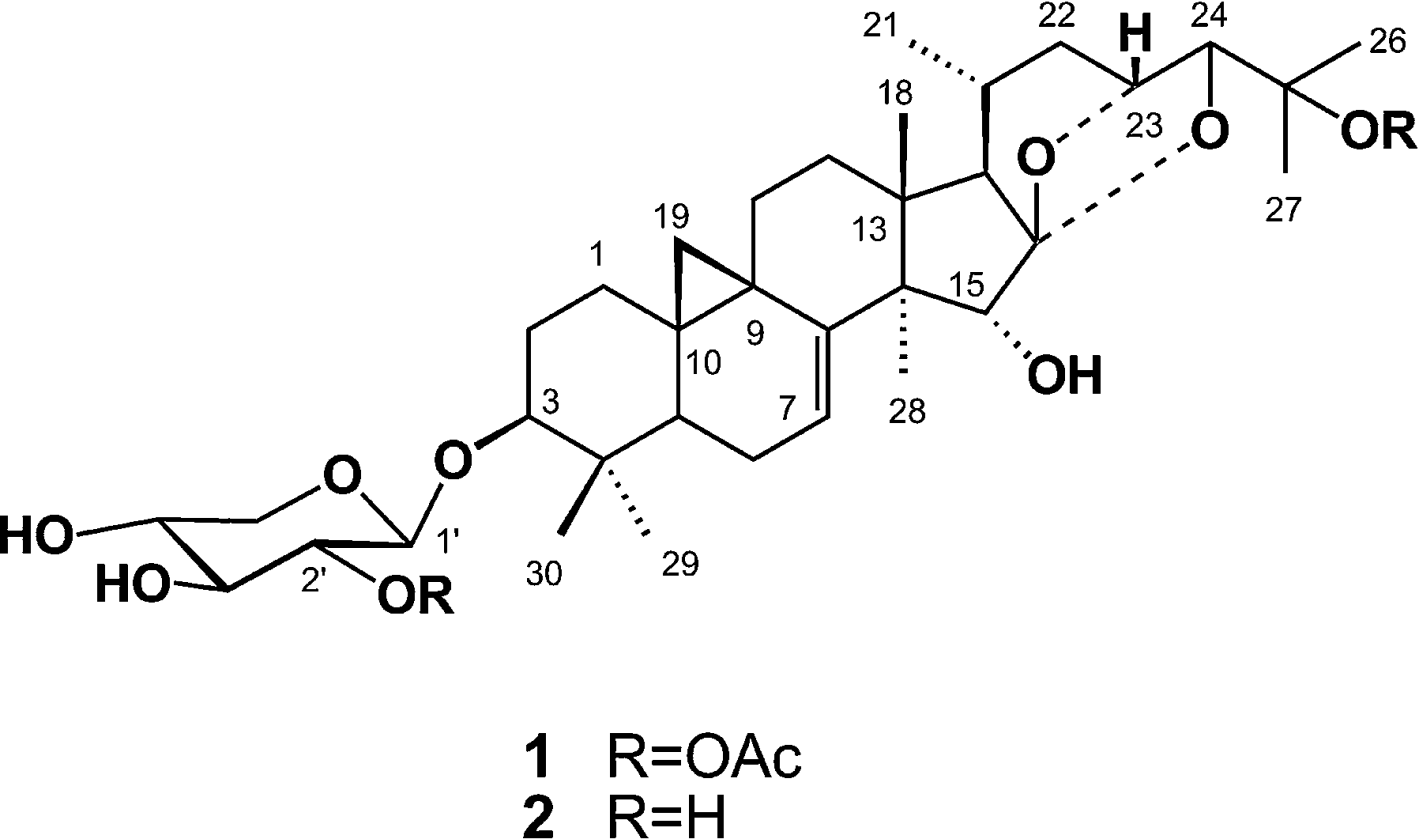
Chemical structures of components ADHC-AXpn and DHC-Xpn

Chinese medicine (CM) is a rich source for the development of anticancer drugs and chemopreventive agents [1]. Cycloartane triterpenoid (CATP) are triterpene glycosides with a unique structural C-19 angular methyl (Fig. 1), are distributed in plants of several genuses, such as *Astragalus*, *Cimicifuga*, and *Thalictrum* [2, 3]. Several CATPs isolated from plants exhibited potential anticancer activities [4–7].

The rhizome of *Cimicifuga foetida* L. (*Sheng ma*), has been used as an anti-inflammatory, analgesic, and antipyretic agent in CM [8]. *C. foetida* contains many CATPs [9, 10], and some CATPs from the *Cimicifuga* genus possessed anti-cancer effects *in vivo* and *in vitro* [4–6]. However, previous research focused on isolation and structure identification rather than biological activity. Our earlier research associated CATP anticancer activity with apoptosis induction and cell cycle arrest in tumor cells [4, 5], but respective biological pathways have not yet investigated.

This study aims to identify any potential novel anticancer CATP from *C. foetida* and evaluate their effects on the growth, colony formation, apoptosis induction and respective pathways in cancer cells.

## Methods

### Plant materials

Rhizome of *C. foetida* was collected in Emei Mountain, Sichuan province, China in October 2012 and identified by Dr. Sibao Chen by comparing its morphological features [8]. A voucher specimen (SZRI20121045) was deposited in the herbarium of state key laboratory of Chinese medicine and molecular pharmacology.

### Equipment

Infrared spectra (IR) were recorded on a Shimadzu IR-450 spectrometer (Shimadzu, Kyoto, Japan). High resolution fast atom bombardment mass spectra (HR-FAB-MS) were recorded on a VG-Autospec-3000 spectrometer (Micromass, Manchester, UK), in *m/z* (percentage relative intensity of base peak). The nuclear magnetic resonance (NMR) spectra were measured in pyridine-*d*_5_ on a Bruker AM-400 spectrometer (BrukerDaltonics, MA, USA), using tetramethyl silane as internal standard. Silica gel-_60H_ (100–200 and 200–300 mesh) and silica gel GF_254_ sheets (0.20–0.25 mm; both from Qingdao Haiyang Chemical Group Co., Qingdao, China) were used for column chromatography (CCG) and thin layer chromatography (TLC), respectively.

### Materials and reagents for pharmacological investigations

3-(4, 5)-Dimethylthiazol-2-yl)-2, 5-diphenyl tetrazolium bromide (MTT), Hoechst 33258, propidium iodide (PI), doxorubicin (Dox) and carbonyl cyanide 3-c hlorophenylhydrazone were purchased from Sigma-Aldrich (St. Louis, MO, USA). 5, 5′, 6, 6′-tetrachloro-1, 1′, 3, 3′-tetraethyl benzimidazolyl-carbocyanine iodide (JC-1) and Alexa Fluor^®^ 488 Annexin V/dead cell apoptosis kit were obtained from invitrogen (Carlsbad, CA, USA). BCA protein assay kit and electro chemiluminescence were obtained from Thermo Fisher Scientific (Rockford, IL, USA). Antibodies against cyclin B1, CDK1, p-CDK1(Thr^161^), caspase-9, cleaved caspase-9, PARP, cleaved PARP, Akt, p-Akt (Thr^308^ and Ser^473^), p-ERK1/2 and p-c-Raf were from cell signaling technology (Beverly, MA, USA). Antibodies against Bcl-2 and Bax were bought from Santa Cruz Biotechnology (Santa Cruz, CA, USA). Protease inhibitor cocktail tablets and phosphatase inhibitor cocktail tablets were supplied by Roche (Roche Applied Science, Mannheim, Germany). Dimethylsulfoxide (DMSO); RPMI-1640 and Dulbecco’s modification of Eagle’s medium (DMEM), phosphate-buffered saline (PBS), fetal bovine serum (FBS), penicillin-streptomycin (P/S) and trypsin-ethylene diamine tetraacetic acid (EDTA) were purchased from life technologies (Grand Island, NY, USA).

### Extraction and isolation

The rhizome of *C. foetida* (0.5 kg) was extracted three times with 95 % EtOH for 1 h under reflux. After combination of extractions and solvent removal, the residue (129 g) was suspended in water (1 L) and partitioned successively with 200 mL each of petroleum ether (60–80 ℃), ethyl acetate and n-BuOH. The ethyl acetate fraction (28 g) was subjected to CCG on silica gel-_60H_ (100–200 mesh). Gradient elution with CHCl_3_–MeOH (1:0, 50:1, 20:1, 10:1 and 0:1), obtained five fractions: A (3.7 g), B (4.9 g), C (6.28 g), D (1.5 g) and E (1.8 g).

Fraction D was subjected to CCG on silica gel-_60H_ (200–300 mesh), eluted with CHCl_3_–MeOH (70:30) and further purified by Sephadex G_10_ CCG, eluted with MeOH to obtain ADHC-AXpn (15 mg).

Fraction C was subjected to repeated CCG on silica gel-_60H_ (200–300 mesh), eluted with CHCl_3_ and acetone (3:1) to give sub-fractions 1–5. Sub-fraction 5 was subjected to CCG on silica gel-_60H_ (200–300 mesh), eluted in gradient with CHCl_3_-MeOH (90:10, 85:15 and 80:20). The fraction eluted by CHCl_3_-MeOH (80:20) was purified by Sephadex G_10_ CCG, and eluted with MeOH, to give DHC-Xpn (40 mg). Chemical structures of ADHC-AXpn and DHC-Xpn were elucidated by their spectral data (IR, MS and ^1^H, ^13^C-NMR) and by comparison with data in the literature. The purity of isolated components was determined over 98 % by peak area normalization method in HPLC analysis by an Agilent 1200 liquid chromatography system (HP Agilent Technologies, Palo Alto, CA, USA).

### Cell culture

Cell lines MCF-7 (estrogen receptor-positive phenotype), HepG2, HeLa and PC3 cells were obtained from American type culture collection (Manassas, VA, USA). HepG2/ADM cells (multidrug resistance phenotype) were kindly provided by Prof. Kwok-Pui Fung (The Chinese University of Hong Kong, Hong Kong, China). Human MCF10A mammary epithelial cells were obtained from Invitrogen (Carlsbad, CA, USA). The MCF-7, HepG2, HepG2/ADM and PC3 cells were cultured in RPMI-1640 medium supplemented with 10 FBS and 1 % (v/v) P/S at 37 ℃ in a humidified incubator containing 5 % CO_2_. HeLa cells were cultured in DMEM medium with the same culture condition mentioned above. Human MCF10A mammary epithelial cells were cultured in a condition mentioned previously [11]. HepG2/ADM cells were cultured with 1.2 μM of Dox; during cell passage to keep their multidrug resistance property as compared with the corresponding parental cells.

### Cell viability assay

The inhibitory effects of ADHC-AXpn and DHC-Xpn on the growth of tested cells were evaluated by MTT assay. Briefly, all tested cells (0.8 × 10^4^/well) were seeded in 96-well plates, cultured for 24 h, then exposed to different concentrations of ADHC-AXpn and DHC-Xpn, respectively for another 24 or 48 h. Subsequently, 30 μL of 5 mg/mL MTT dissolved in PBS was added to each well and incubated for 4 h after removing the media. Formazan crystals were dissolved with 100 μL of DMSO, with absorbance at 490 nm measured by Bio-Rad 680 microplate reader (Bio-Rad, CA, USA). Cell viability was determined as a percentage of the control, with Dox as positive control.

### Colony formation assay

MCF-7 cells (2.5 × 10^5^/well) were seeded in 6-well plates, cultured for 24 h, then exposed to different concentrations of ADHC-AXpn (20, 30 or 35 μM) for 48 h before digestion in 0.25 % trypsin to reconstitute single-cell suspensions. Cell suspensions were then transferred into 6-well plates (600 cells/well) and incubated at 37 ℃ for 12 days. The formed colonies were fixed in 4 % paraformaldehyde for 30 min and stained with 1 % crystal violet for 5 min. These experiments were performed three times. Colonies with diameters larger than 0.5 mm were counted to calculate the colony formation rate.

### Hoechst 33258 staining assay

The nuclear morphology of cells treated with ADHC-AXpn was observed with a Hoechst 33258 staining assay. MCF-7 cells (2.5 × 10^5^/well) were seeded in 6-well plates, cultured for 24 h, and treated with different concentrations of ADHC-AXpn (20, 30 or 35 μM) for 48 h. The cells were then washed three times with PBS, fixed in 4 % paraformaldehyde at 4 ℃ for 30 min, and stained with 10 μg/mL of Hoechst 33258 for 15 min at 37 ℃. Changes in nuclear morphology were monitored with an Olympus IX51 inverted microscope (Olympus Corporation of the Americas, Inc., Central Valley, PA, USA).

### Cell cycle analysis

Cell cycle distribution was analyzed by a fluorescent probe propidium iodide (PI) staining assay. MCF-7 cells (2.5 × 10^5^/well) were seeded in 6-well plates, cultured for 24 h, then treated with different concentrations of ADHC-AXpn (20, 30 or 35 μM) for 24 or 48 h. Cells were then collected and fixed with 75 % ethanol at 4 ℃ overnight. After centrifugation in an Eppendorf 5417R centrifuge (Eppendorf Corp., Hamburg, Germany) at 800*g* for 5 min, the cells were incubated with PBS containing 0.02 mg/mL PI and 0.1 mg/mL RNase A in darkness at 37 ℃ for 30 min. The emitting fluorescence was measured by Guava Easy Cytometer (Guava Technologies, Millipore, Billerica, MA, USA); data were analyzed with MultiCycle AV software (Phoenix Flow Systems, San Diego, CA, USA).

### Annexin V-FITC/PI double staining assay for apoptosis

MCF-7 cells (2.5 × 10^5^/well) were seeded in 6-well plates, cultured for 24 h, and exposed to differing concentrations of ADHC-AXpn (20, 30 or 35 μM) for 36 h. Cells were then collected and stained by Alexa Fluor^®^ 488 Annexin V/dead cell apoptosis kit according to manufacturer instructions. Green fluorescence emitted from Annexin V-FITC and red fluorescence from PI were detected by Guava Easy Cytometer (Guava Technologies, Millipore, Billerica, MA, USA). Data were analyzed by FlowJo 7.6 software (TreeStar, SanCarlos, CA, USA).

### Measurement of mitochondrial membrane potential (MMP)

Changes in MMP were measured using a lipophilic cationic fluorescent probe JC-1. Briefly, MCF-7 cells (2.5 × 10^5^/well) were seeded in 6-well plates, cultured for 24 h, then treated with ADHC-AXpn (20, 30 or 35 μM) for 24 h. The cells were collected and incubated with 5 μM JC-1 in darkness at 37 ℃ for 15 min, then assayed with a Guava Easy Cytometer (EasyCyte 8 HT, Millipore, USA) to evaluate changes in MMP..

### Western blotting assay

MCF-7 cells (2.5 × 10^5^/well) were seeded in 100-mm culture dishes, cultured for 24 h, then incubated with differing concentrations of ADHC-AXpn for various times. Cells were then collected by trypsinization with 0.25 % Trypsin-EDTA and transferred to centrifuge tubes. After centrifugation at 800*g* for 5 min, cells were washed twice with ice-cold PBS and then lysed in RIPA buffer containing 0.5 M DTT, 0.1 M PMSF and 20× phosphatase inhibitor for 30 min on ice. Protein samples were harvested by centrifugation at 12,000*g* at 4 ℃ for 12 min. Protein contents were quantified with BCA Protein Assay Kit (Thermo Scientific Pierce, Rockford, USA). The protein samples (35 μg) along with a rainbow-colored protein molecular marker (Amersham, Buckinghamshire, UK) were separated by 10 % SDS-PAGE and transferred to polyvinylidene fluoride (PVDF) membranes (Millipore, Bedford, MA, USA) by a semi-dry transfer apparatus (Bio-Rad, CA, USA). The membranes were blocked with 5 % nonfat dry milk in Tris-buffered saline containing 0.1 % Tween-20 (TBS-T) at room temperature for 1 h and incubated overnight with primary antibodies containing 5 % BSA at 4 ℃. Membranes were washed with TBS-T three times and appropriate secondary antibodies conjugated with horseradish peroxidase were added and incubated for another hour. Finally, immunoblots were visualized by enhanced chemiluminescence detection reagents, with β-actin as a loading control.

### Statistical analysis

Each experiment was performed for three times and data were presented as mean ± standard deviation (SD). Analysis of variance (ANOVA) was performed to detect significant differences. In multiple comparisons, Tukey post-test was used. *P* value less than 0.05 was considered statistically significant. Analyses of dose-, concentration- and time-dependent effects were performed by sigmoidal non-linear regression. All statistical analysis tests were performed by Graph Pad Prism 5.0 for Windows (GraphPad Software, San Diego California, USA).

## Results

### Structure elucidation of ADHC-AXpn and DHC-Xpn

**Fig. 2:**
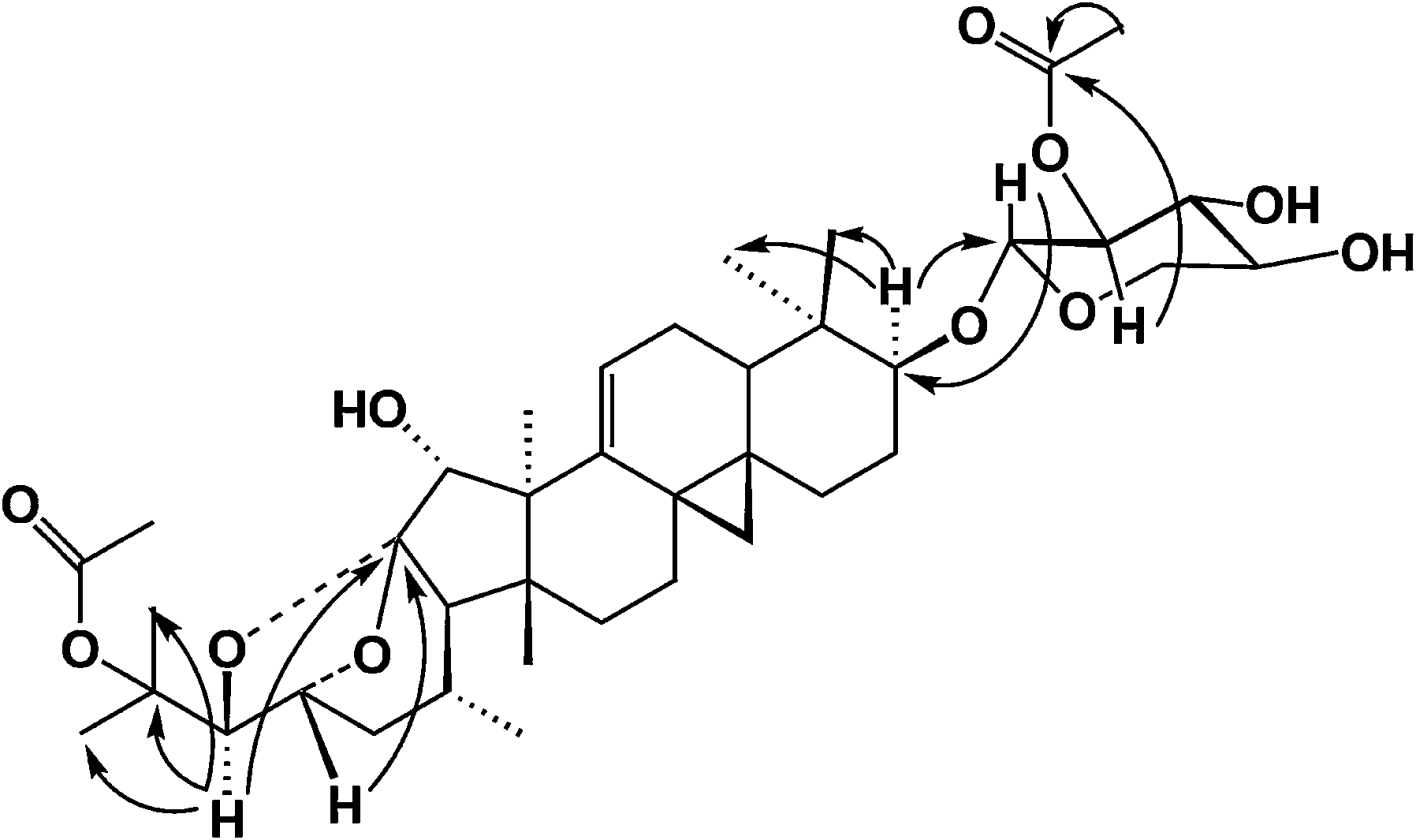
Key HMBC for ADHC-AXpn

ADHC-AXpn was isolated as a white powder. The HR-FAB-MS showed a pseudo-molecular ion peak at m/z 725.3887 [M + Na]^+^ (calc. 725.3893), which supported the molecular formula as being C_39_H_58_O_11_. The IR spectrum displayed the presence of hydroxyl groups (3338 cm^−1^) and a carbonyl group (1730 cm^−1^). The ^1^H-NMR spectrum (Table 1) showed signals of cyclopropane methylene groups at δ 0.43 and 0.96 ppm (each ^1^H, d, *J* = 4.0 Hz), six methyl groups at δ 1.05, 1.08, 1.13, 1.20, 1.65 and 1.67 ppm; an aromatic proton at δ 4.82 ppm (d, *J* = 8.0 Hz), and a series of overlapped signals, which suggested that ADHC-AXpn was a cycloartane-type triterpene glycoside [12]. The ^13^C-NMR (Table 1) displayed the diagnostic signals of two oxygen-bearing alkyl carbons at δ 87.0 (C-24) and 72.1 ppm (C-23), and an oxygen-bearing quaternary carbon at δ 113.0 ppm (C-16), which implied that ADHC-AXpn was a cimigenol-type triterpene [13]. By fully comparing ^1^H and ^13^C NMR spectral data of ADHC-AXpn with those of the known component 25-*O*-acetyl-7,8-didehydrocimigenol-3-*O*-*β*-d-xylopyranoside (25-AC) [12], the structure of ADHC-AXpn was similar to that of 25-AC, excepting an additional acetyl moiety. When the ^13^C-NMR spectrum of ADHC-AXpn was compared with that of 25-AC, the signals of C-1′ and C-3′ were seen to shift to higher field by 2.6 and 2.0 ppm, while the signal of C-2′ shifted to lower field. These chemical shift variations suggested an acetyl moiety was linked to the C-2′ of the xylose unit [12]. Locations of the two acetyl groups were directly confirmed by hetero-nuclear multiple bond correlation (HMBC) experiments (Fig. 2). Briefly, significant correlation was observed between H-2′ (δH 5.56 ppm, dd, *J* = 7.5, 8.1 Hz) and the carbonyl signal at δ170.3 ppm, which indicated an acetyl group was assignable to C-2′. The methyl signals at δH 1.65 ppm (Me-26)/δH 1.67 ppm (Me-27) also showed correlations with a quaternary carbon signal at δC 83.44 ppm (C-25), the methine carbon signal at δC 87.05 ppm (C-24), and the methyl carbon signals at δC 21.58 ppm (C-27)/δC 24.14 ppm (C-26), which indicated that another acetyl group was located at C-25. A complete ^1^H and ^13^C-NMR spectral assignment of ADHC-AXpn was summarized in Tables 1 and 2.

**Table 1:** ^1^H and ^13^C-NMR (400 MHz) data of ADHC-AXpn in pyridine-*d*_5_

Position	δ_H_ (*J* = Hz)	δ_C_	Position	δ_H_ (*J* = Hz)	δ_C_
1	1.20 m, 1.51 m	30.43	20	1.57 m	23.67
2	1.87 m, 2.26 m	29.67	21	0.89 d (6.5)	19.95
3	3.38 dd (4.0, 11.9)	88.61	22	0.94 m, 2.26 m	38.07
4		40.37	23	4.60 d (9.3)	72.17
5	1.28 m	42.78	24	4.11 s	87.05
6	1.59 m, 1.90 m	22.58	25	–	83.44
7	6.10 dd (1.3, 7.5)	113.01	26	1.65 s	24.14
8	–	148.22	27	1.67 s	21.58
9	–	21.95	28	1.13 s	18.74
10	–	28.60	29	1.20 s	25.79
11	1.13 m, 2.04 m	25.71	30	1.05 s	14.35
12	1.53 m, 1.62 m	34.31	1′	4.84 d (7.5)	104.81
13	–	41.49	2′	5.56 dd (7.5, 8.1)	75.91
14	–	50.74	3′	4.13 dd (8.1, 8.1)	76.51
15	4.27 s	78.44	4′	4.21 m	71.60
16	–	114.4	5′	3.68 dd (10.0, 11.0) 4.31dd (5.0, 10.5)	67.41
17	1.49 d (10.0)	59.51			
18	1.08 s	21.78	25-COCH_3_	1.95 s	170.4, 21.5
19	0.43 d (4.0),0.96 d (4.0)	28.35	2′-COCH_3_	2.14 s	170.3, 21.5

**Table 2:** The inhibitory effects of ADHC-AXpn and DHC-Xpn on the viability of human carcinoma cells

	ADHC-AXpn (IC_50_, μM)		DHC-Xpn (IC_50_, μM)		Dox
	24 h	48 h	24 h	48 h	48 h
MCF-7	30.32 ± 0.79	27.81 ± 1.29	>50	42.14 ± 4.63	4.07 ± 1.33
HepG2	40.73 ± 1.46	35.65 ± 1.17	>50	42.51 ± 2.57	3.80 ± 0.95
HepG2/ADM	33.66 ± 3.64	29.61 ± 0.26	>50	>50	143.47 ± 4.76
HeLa	41.25 ± 0.23	35.73 ± 2.15	>50	>50	4.62 ± 1.37
PC3	>50	>50	>50	>50	5.52 ± 1.19

Taken together, the structure of ADHC-AXpn was formulated as 25-*O*-acetyl-7,8-didehydrocimigenol-3-*O*-*β*-d-(2-acetyl) xylopyranoside (Fig. 1). The known DHC-Xpn was determined as 7,8-didehydrocimigenol-3-*O*-*β*-d-xylopyranoside by comparison of its spectral data with those reported in the literature [14].

### Growth inhibition effects of ADHC-AXpn and DHC-Xpn on carcinoma cells

**Fig. 3:**
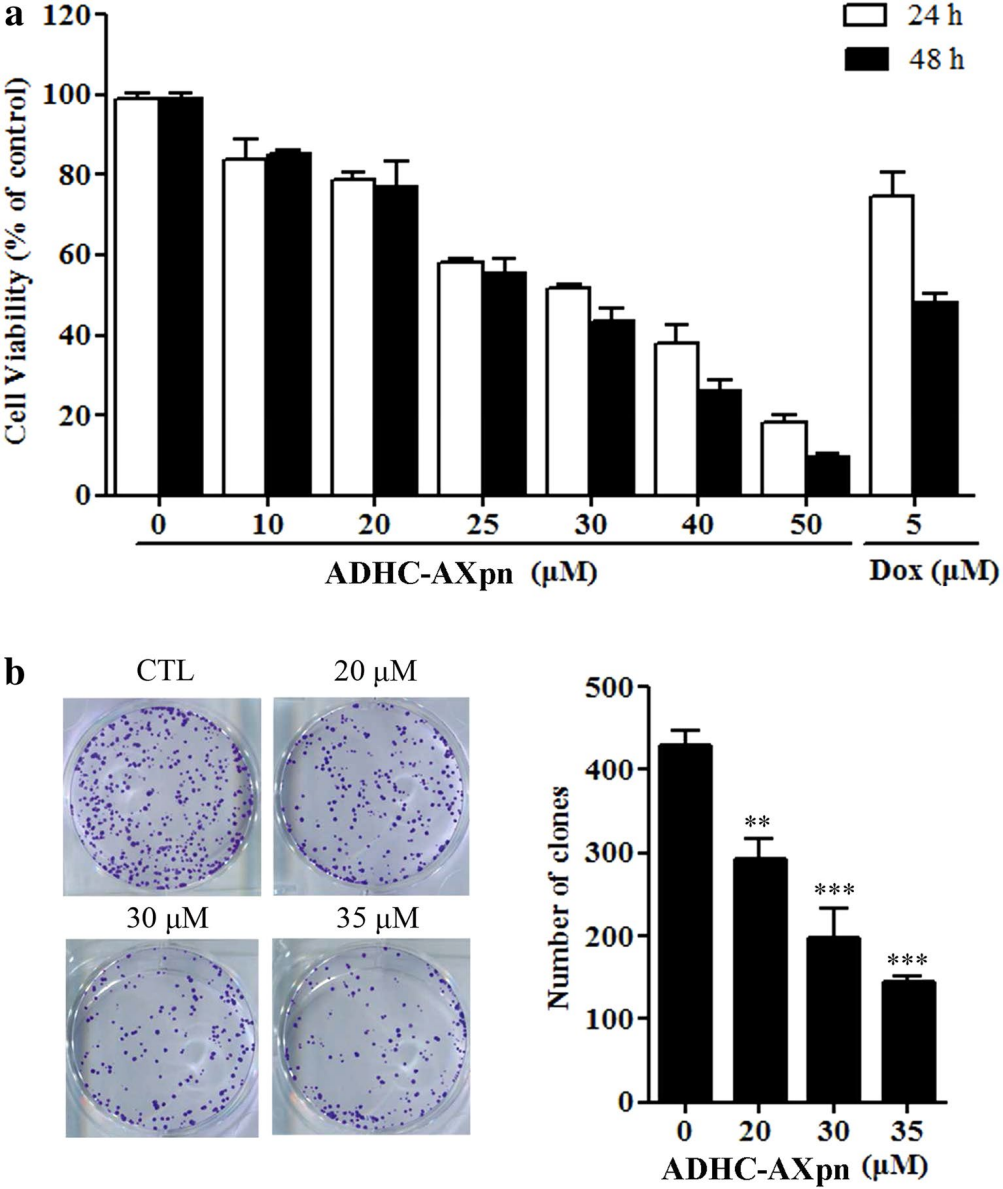
Growth inhibitory activity of ADHC-AXpn in MCF-7 cells. **a** Cells were treated with increasing concentrations of ADHC-AXpn (10 to 50 μM) for 24 and 48 h; cell survival was then assessed by MTT assay. Doxorubicin (Dox) exposure at 5 μM was used as a positive control. **b** After exposure to ADHC-AXpn at concentrations of 20, 30 or 35 μM, cells were seeded onto 6-well plates (600 cells/well) for assessing colony formation. Data are expressed as mean ± SD of three independent experiments with **P = 0.0078; ***P < 0.001

The inhibitory effects of ADHC-AXpn and DHC-Xpn on the viability of various carcinoma cells were determined by MTT assay. ADHC-AXpn was much more potent than DHC-Xpn in decreasing the viability of the tested cells (MCF-7, HepG2/ADM, HepG2, HELA and PC3 cells) in both 24 and 48 h treatment periods apart from PC3 cells (Table 2). Among these cancer cells treated with ADHC-AXpn, MCF-7 cells appeared to be more sensitive to ADHC-AXpn than other cancer cells, as indicated by the IC_50_ values (Fig. 3a). As shown in Fig. 3b, the colony formation assay also confirmed that ADHC-AXpn (20, 30 or 35 μM) could inhibit proliferation of MCF-7 cells (*P* = 0.0078, *P* < 0.001, *P* < 0.0001). The IC_50_ values of ADHC-AXpn in human MCF10A mammary epithelial cells at 48 h were 78.63 ± 3.26 μM, which was much higher than for cancer cell line MCF-7. These data indicated that ADHC-AXpn was selectively cytotoxic for cancer cells.

### ADHC-AXpn induces cell cycle arrest in MCF-7 cells

**Fig. 4:**
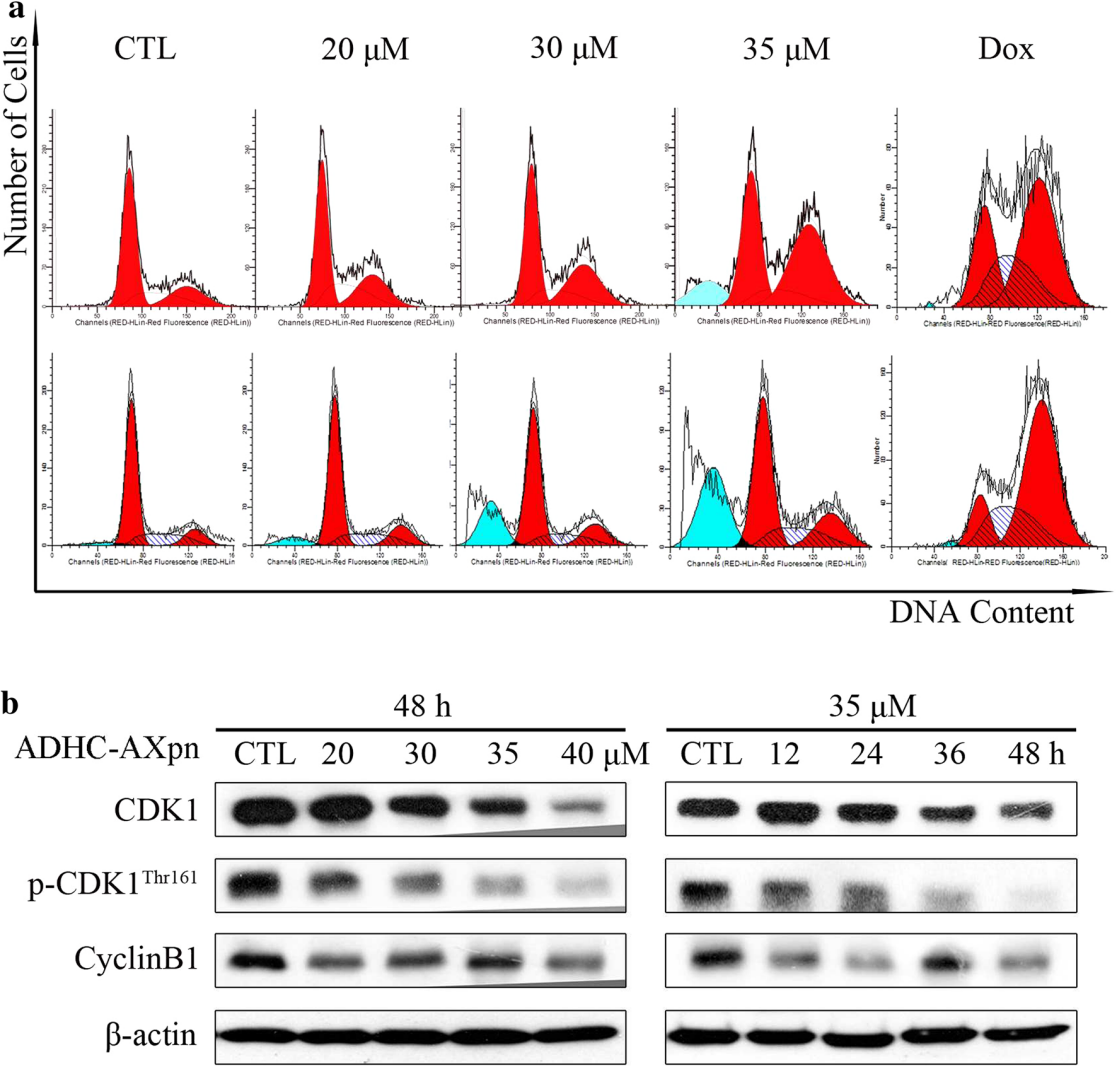
Cell-cycle arrest induced by ADHC-AXpn in MCF-7 cells. **a** Cell cycle profiles of MCF-7 cells after treatment with ADHC-AXpn for 24 or 48 h. Cells exposed to indicated concentrations of ADHC-AXpn were collected, fixed with 75 % ethanol overnight, and stained with PI for 30 min; cell-cycle profiles were then determined by flow cytometry. Doxorubicin (dox) exposure at 5 μM was used as a positive control. **b** Changes in cell-cycle regulator protein expression after exposure to ADHC-AXpn in MCF-7 cells. After treatment with ADHC-AXpn, total cell lysates were immunoblotted to detect CDK1, p-CDK1 (Thr^161^) and cyclin B1 expression levels, using specific antibodies. β-actin served as a loading control

We investigated the effect of ADHC-AXpn on the MCF-7 cell cycle by flow cytometry. Treating MCF-7 cells with ADHC-AXpn for 24 h induced a G_2_/M arrest (Fig. 4a). The percentage of cells in subG_1_ phase increased when ADHC-AXpn exposure was extended to 48 h. To explore the mechanism of ADHC-AXpn-induced G_2_/M arrest, we investigated its effects on G_2_/M regulatory proteins. Western blotting showed ADHC-AXpn caused a time- and dose-dependent decrease in cyclin B1 (Fig. 4b). ADHC-AXpn decreased cyclin-dependent kinase 1 (CDK1), which regulates the cell cycle by forming the CDK-cyclin complex, and phospho-CDK1 (Thr^161^). ADHC-AXpn arrests the cell cycle in G_2_/M phase by decreasing expression of cyclin B1, CDK1 and phospho-CDK1 (Thr^161^).

### Induction of apoptosis by ADHC-AXpn in MCF-7 cells

**Fig. 5:**
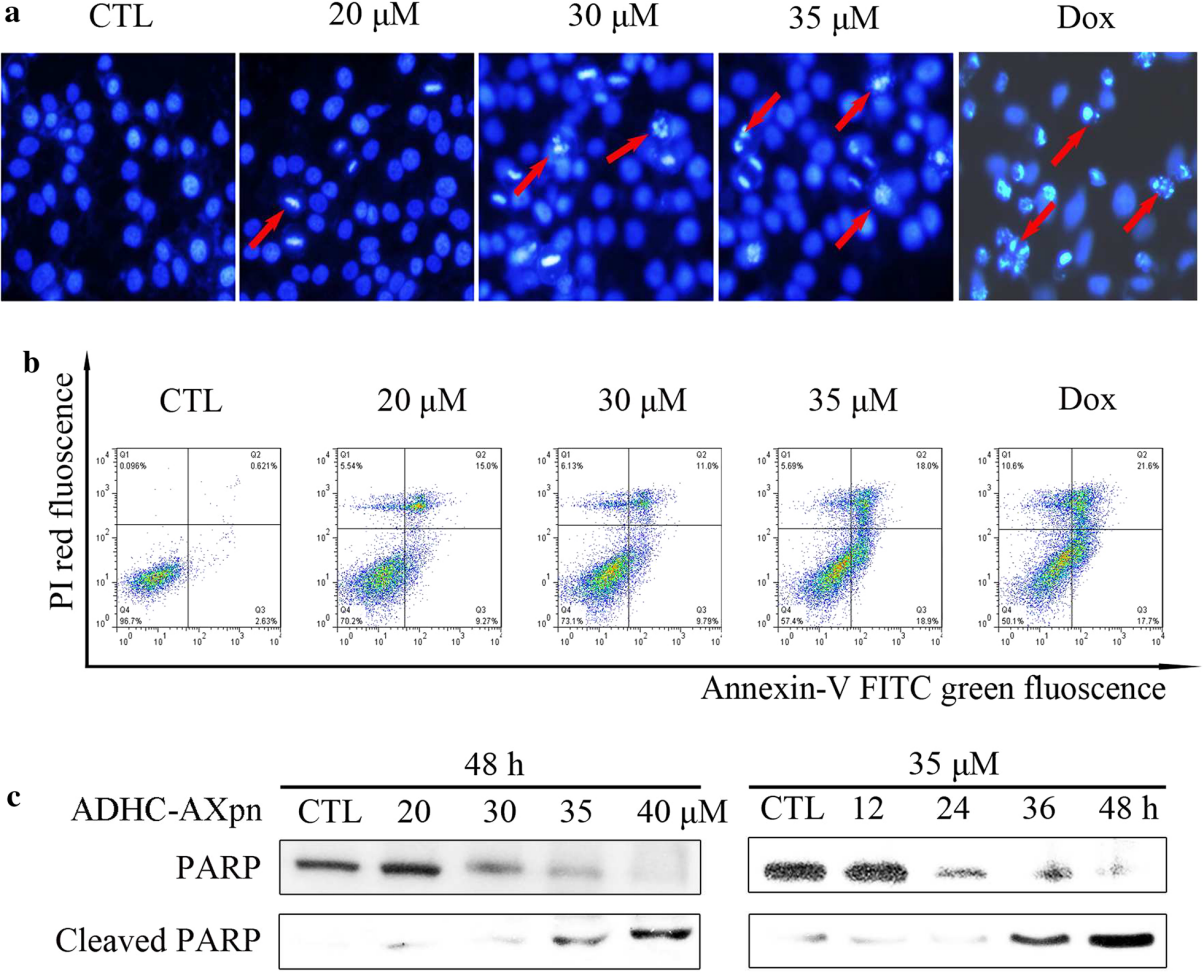
Apoptosis induced by ADHC-AXpn in MCF-7 cells. **a** Representative image of MCF-7 cells stained with Hoechst 33258 after treatment with ADHC-AXpn for 48 h, under fluorescence microscope; *red arrows*: cell shrinkage, nuclear fragmentation. **b** Apoptosis quantification, using Annexin V/PI double staining assay after treatment with ADHC-AXpn for 36 h. MCF-7 cells were harvested and stained with PI and Annexin V-FITC in darkness for 15 min, followed by flow cytometry analysis.* Lower right*: Annexin V^+^/PI^−^ (early apoptosis);* upper right*: Annexin V^+^/PI^+^ (late apoptosis). **c** *Western blot* showing PARP cleavage in MCF-7 cell lysates after treatment with ADHC-AXpn, using indicated antibodies. Loading control: β-actin; positive control: doxorubicin (Dox), 5 μM

Changes of cellular morphology were examined by fluorescent microscopy analysis (Fig. 5a). After treatment with different concentrations of ADHC-AXpn (20, 30 or 35 μM) for 48 h, cells stained with Hoechst 33258 showed apoptotic features, such as cytoplasmic shrinkage and condensation of nuclear chromatin. Consistently, PI/Annexin V double staining analysis (Fig. 5b) showed that treatment with ADHC-AXpn (30 or 35 μM) for 48 h led to significantly increased cell percentages in early and late apoptotic phases (*P* = 0.0094 and *P* = 0.0042 vs. control, respectively). Cleavage of PARP was also seen in ADHC-AXpn-treated MCF-7 cells (Fig. 5c).

### Activation of mitochondrial apoptotic pathway and inhibition of Akt phosphorylation

**Fig. 6:**
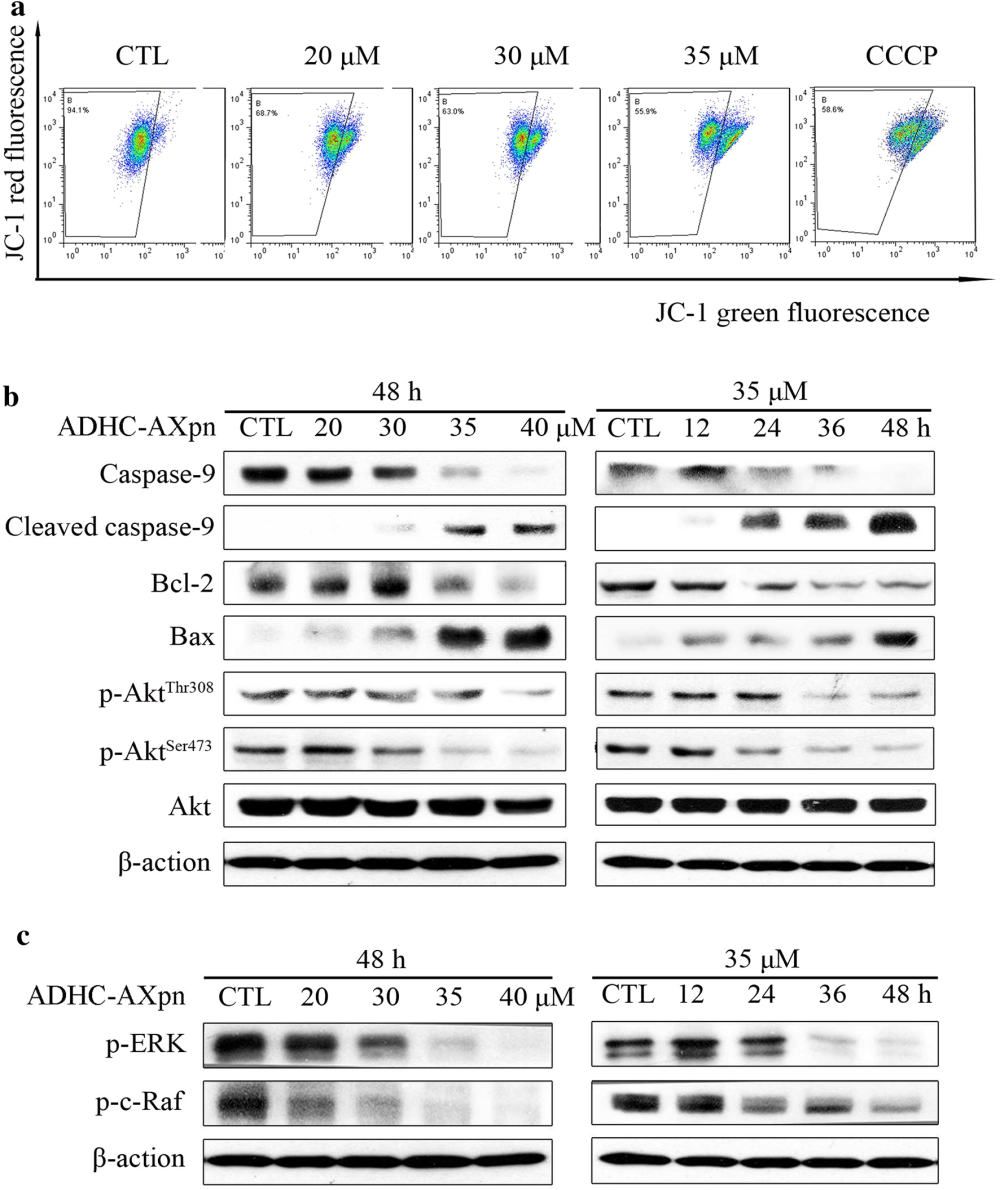
Effect of ADHC-AXpn on mitochondrial function and Raf/MEK/ERK signaling pathway in MCF-7 cells. **a** Flow cytometry shows mitochondrial membrane depolarization induced by treatment with indicated concentrations of ADHC-AXpn for 24 h; detached cells were stained with JC-1; generic mitochondrial membrane depolarizer CCCP (20 μM), used as positive control, showed an expected decrease in red fluorescence. **b** *Western blot* shows caspase activation and changes in expression levels of Bcl-2 family proteins and Akt proteins in MCF-7 cells after treatment with ADHC-AXpn, using specific antibodies against caspase-9, cleaved caspase-9, Bcl-2 and Bax, Akt and p-Akt (Thr^308^ and Ser^473^), respectively. **c** *Western blot* shows inhibition of Raf/MEK/ERK signaling pathway related proteins in MCF-7 cells after treatment with ADHC-AXpn, using the indicated antibodies; loading control: β-actin

Changes of MMP were measured by a fluorescent dye (JC-1) to further investigate the apoptotic pathway. MCF-7 cells treated with ADHC-AXpn (20, 30 or 35 μM) for 24 h showed a gradual decrease in the percentage of cells with high MMP from 94.1 (control) to 68.7 %, 63.0 and 55.9 %, respectively (Fig. 6a). We examined expression levels of pro-apoptotic protein Bax and anti-apoptotic protein Bcl-2. Western blot analysis showed that ADHC-AXpn treatment down-regulated Bcl-2 and clearly up-regulated Bax (Fig. 6b). The precursor form of caspase-9 was decreased, whereas expression of cleaved caspase-9 was increased. ADHC-AXpn exposure remarkably decreased p-Akt (Thr^308^ and Ser^473^), although total Akt showed no visible change.

### Raf/MEK/ERK pathway inhibition by ADHC-AXpn in MCF-7 cells

The Raf/MEK/ERK pathway is beneficial for survival of MCF-7 cells, and blockage of this pathway can trigger apoptosis [15]. To investigate whether the Raf/MEK/ERK pathway affects ADHC-AXpn-induced apoptosis, we assessed expression levels of p-ERK1/2 and p-c-Raf. Compared with the control group, ADHC-AXpn treatment decreased phosphorylation of ERK1/2 in both time- and dose-dependent manners (Fig. 6c). The upstream regulator of ERK1/2, p-c-Raf, was also down-regulated after exposure to ADHC-AXpn.

## Discussion

In the present study, a novel cycloartane triterpenoid, ADHC-AXpn and the known DHC-Xpn were isolated and identified from *C. foetia*. The cytotoxic effects of these two isolated components on human cancer cells MCF-7, HepG2, HepG2/ADM, HeLa, PC3 and normal human mammary epithelial cells MCF10A were assessed by MTT assay and colony formation assay. ADHC-AXpn, which possesses acetyl groups at C-25 and C-2′ in its sugar groups, was more cytotoxic toward all tested cell lines than was DHC-Xpn, which bears hydroxyl groups at C-25 and C-2′ in its sugar group. Addition of a hydrophobic group (such as acetyl or halogen) linked to C-25 or other positions remarkably increased cytotoxic activity, which were consistent with a previous report on the structure–activity relationship of cycloartane triterpenoids in other cells [4]. The IC_50_ value of ADHC-AXpn for normal human mammary epithelial cells MCF10A was higher than those for cancer cells.

Activation of the apoptotic pathway in tumor cells is a major protective mechanism against the development and progression of cancer [16]. On the basis of the detected cytotoxic effects of ADHC-AXpn and DHC-Xpn on MCF-7, HepG2, HepG2/ADM, HeLa and PC3 cells (Table 2), further studies were performed to explore the mechanism of ADHC-AXpn-induced cell death in MCF-7 cells. To our knowledge, our study was the first one to demonstrate that ADHC-AXpn could induce MCF-7 cell death *via* apoptosis, accompanied with stereotypical apoptotic features that include cell shrinkage, phosphatidylserine (PS) externalization and PARP cleavage. ADHC-AXpn inhibited cyclin B1 and CDK1, and thereby led to clear G_2_/M arrest.

Apoptosis has two major pathways: the cell death receptor-mediated apoptotic pathway and the mitochondrial-mediated apoptotic pathway [17]. The latter is also referred to as the intrinsic apoptosis pathway, and it is involved in the caspase-dependent apoptotic pathway [18, 19]. Bcl-2 family proteins are major regulators of the mitochondrial pathway; Bcl-2 is a major anti-apoptosis protein, integrated in the mitochondrial membrane, and Bax is inactive in the cytosol or loosely attached to intracellular membranes as a monomer [20, 21]. The over-expression of Bax and lower-expression of Bcl-2 induce the MMP collapse and a subsequent cascade activation of caspases through homologous dimerization, and eventually lead to apoptosis. Four isolated cycloartane triterpenoids from *Cimicifuga yunnanensis*, another species of *Cimicifuga genus*, 25-*O*-acetylcimigenol-3-*O*-β-d-xylopyranoside, 25-chlorodeoxycimigenol-3-*O*-β-d-xylopyranoside, 25-*O*-acetylcimigenol-3-*O*-α-l-arabinopyranoside and 23-*O*-acetylcimigenol-3-*O*-β-d-xylopyranoside increased expression of p53 and Bax, resulting in mitochondria-mediated apoptosis [6]. Our results showed similar phenomena, including changes in Bcl-2 and Bax levels, and decreased MMP and caspase-9 activation (Fig. 6a, b), implying that mitochondrial dysfunction was involved in ADHC-AXpn-induced apoptosis in MCF-7 cells. In addition, decreased Akt phosphorylation might be involved in ADHC-AXpn-induced apoptosis, as Akt activation protects cells from apoptosis via inhibiting pro-apoptotic molecues [22].

The Raf/MEK/ERK pathway can transduce signals that originate from growth factor receptors to transcription factors, thus controlling cell survival and proliferation [15]. Raf, mediated cell cycle progression by regulating cell-cycle associated proteins such as Cdks, cyclins and p21^Cip1^ [23, 24]. The Raf/MEK/ERK pathway also affects apoptosis progression. Raf can induce transcription of anti-apoptotic genes such as *Bcl*-2, and activation of the Raf/MEK/ERK pathway, leading to phosphorylation and inactivation of the pro-apoptotic protein Bad [25–27]. ERK1/2 is required for survival signaling and ERK1/2 inhibition is a crucial part of the apoptotic mechanisms of various stimuli [28]. Our present studies on the regulatory effects of ADHC-AXpn on cyclin B1, CDK1, caspase-9, Bax and Bcl-2, suggested that the Raf/MEK/ERK pathway might be involved in ADHC-AXpn-induced apoptosis.

## Conclusions

ADHC-AXpn significantly suppressed the growth of MCF-7 cells, induced mitochondrial apoptosis and cell-cycle arrest, and inhibited Raf/MEK/ERK signaling pathway and Akt phosphorylation.

## Abbreviations

*Sheng ma*: the rhizome of *Cimicifuga foetida* L
CM: Chinese medicine
ADHC-AXpn: 25-*O*-acetyl-7,8-didehydrocimigenol-3-*O*-*β*-d-(2-acetyl)xylopyranoside
DHC-Xpn: 7,8-didehydrocimigenol-3-*O*-*β*-d-xylopyranoside
25-AC: 25-*O*-acetyl-7,8-didehydrocimigenol-3-*O*-*β*-d-xylopyranoside
CATP: cycloartane triterpenoid
IR: infrared spectra
HR-FAB-MS: high resolution fast atom bombardment mass spectra
NMR: nuclear magnetic resonance
HMBC: hetero-nuclear multiple bond correlation
CCG: column chromatography
TLC: thin layer chromatography
MTT: 3-(4, 5)-Dimethylthiazol-2-yl)-2, 5-diphenyl tetrazolium bromide
PI: propidium iodide
Dox: doxorubicin
JC-1: 5, 5′, 6, 6′-tetrachloro-1, 1′, 3, 3′-tetraethyl benzimidazolyl-carbocyanine iodide
DMSO: dimethyl sulfoxide
FBS: fetal bovine serum
PBS: phosphate buffered saline
PI: peromide iodine
P/S: penicillin-streptomycin
